# MARITAL QUALITY AND LONELINESS AMONG AGING COMBAT VETERANS: THE MODERATING ROLE OF PTSD SYMPTOMS

**DOI:** 10.1093/geroni/igac059.599

**Published:** 2022-12-20

**Authors:** Christina Marini, Jeremy Yorgason, Anica Pless Kaiser

**Affiliations:** Adelphi University, Garden City, New York, United States; Brigham Young University, Provo, Utah, United States; VA Boston Medical Center, Jamaica Plain, Massachusetts, United States

## Abstract

Loneliness is a robust predictor of aging veterans’ health. Even married older adults may experience loneliness if their relationships are of poor quality. We therefore examined facets of marital quality as predictors of loneliness within a sample of aging veterans: (1) companionship (relationship promotes connection to spouse) and (2) sociability (relationship promotes connection to others). We further evaluated whether veterans’ PTSD symptoms moderated these associations. We utilized two waves of data from 269 Vietnam-era combat veterans (M age = 60.5, SD = .73) collected in 2010 and 2016. Upon controlling for baseline loneliness, demographics, and chronic conditions, higher companionship and sociability each predicted lower subsequent loneliness. We detected interactions between companionship and PTSD subclusters. For example, companionship protected against loneliness only for veterans with low and moderate (but not high) avoidance. Findings highlight nuances in how marital quality predicts aging veterans’ loneliness, some of which are dependent on PTSD symptoms.

